# A Topology-Centric View on Mitotic Chromosome Architecture

**DOI:** 10.3390/ijms18122751

**Published:** 2017-12-18

**Authors:** Ewa Piskadlo, Raquel A. Oliveira

**Affiliations:** Instituto Gulbenkian de Ciência, Rua da Quinta Grande 6, 2780-156 Oeiras, Portugal; episkadlo@igc.gulbenkian.pt

**Keywords:** mitotic chromosomes, chromosome condensation, mitosis, topoisomerase II, condensin, cohesin, ultra-fine bridges, sister chromatid resolution, catenation, sister chromatid intertwines

## Abstract

Mitotic chromosomes are long-known structures, but their internal organization and the exact process by which they are assembled are still a great mystery in biology. Topoisomerase II is crucial for various aspects of mitotic chromosome organization. The unique ability of this enzyme to untangle topologically intertwined DNA molecules (catenations) is of utmost importance for the resolution of sister chromatid intertwines. Although still controversial, topoisomerase II has also been proposed to directly contribute to chromosome compaction, possibly by promoting chromosome self-entanglements. These two functions raise a strong directionality issue towards topoisomerase II reactions that are able to disentangle sister DNA molecules (in trans) while compacting the same DNA molecule (in cis). Here, we review the current knowledge on topoisomerase II role specifically during mitosis, and the mechanisms that directly or indirectly regulate its activity to ensure faithful chromosome segregation. In particular, we discuss how the activity or directionality of this enzyme could be regulated by the SMC (structural maintenance of chromosomes) complexes, predominantly cohesin and condensin, throughout mitosis.

## 1. Introduction

**Figure 1:**
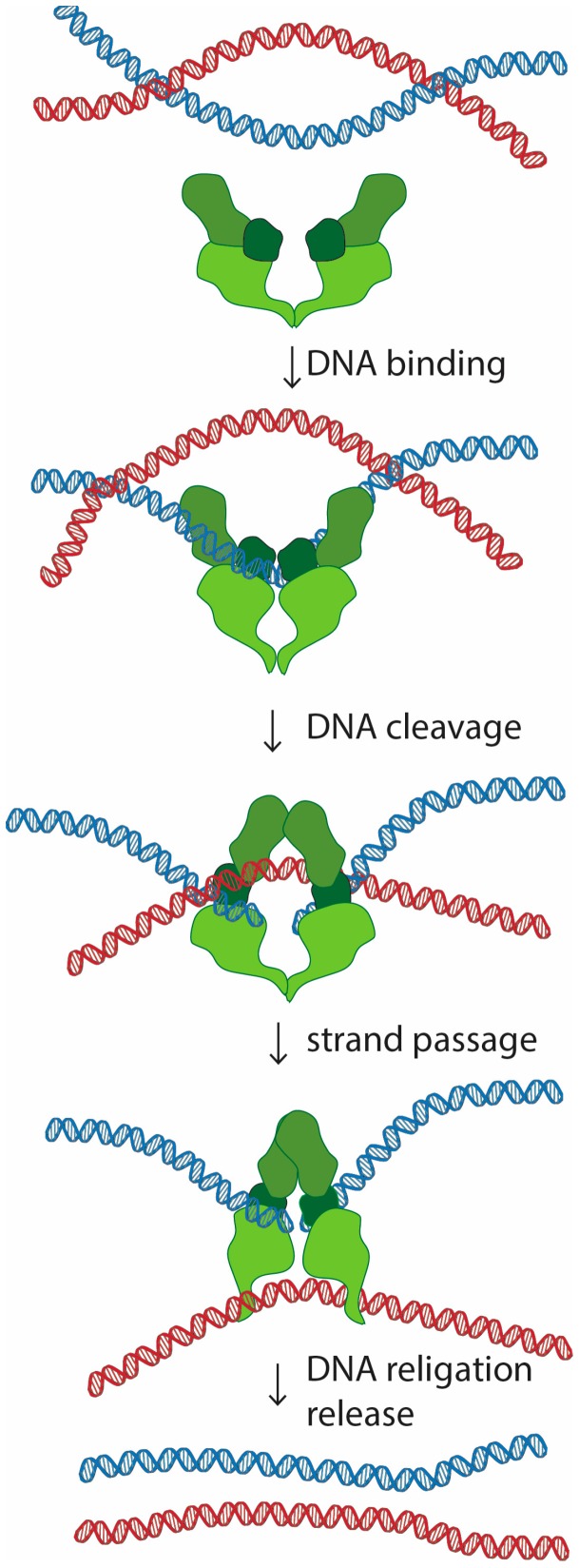
Mechanism of DNA decatenation by topoisomerase II. Topoisomerase II binds to one of the entangled (catenated) DNA molecules. The reaction involves cleavage of the bound DNA strand (blue), introducing a double strand break. Topoisomerase II remains covalently connected to the cut DNA, preventing its dissociation. Once the continuity of one of the DNA strands is severed, topoisomerase II can transport the other DNA molecule (marked in red) through the created gap. Upon strand passage, the cleaved DNA is ligated back together, and topoisomerase II releases the DNA molecule.

Topoisomerase II is a homodimer that performs a unique role in living cells. Besides the ability to change the supercoiling state of DNA that it shares with other types of topoisomerases, only topoisomerase II can untangle topologically intertwined DNA molecules (catenations). These reactions are accomplished through a strand-passing activity, in which one double-stranded DNA segment passes through a transient double-strand break in another DNA molecule (Figure 1). Notability, evidence in vitro suggests that this reaction is reversible, and topoisomerase II is able to release, but also introduce new entanglements between DNA molecules [1,2,3].

**Figure 2:**
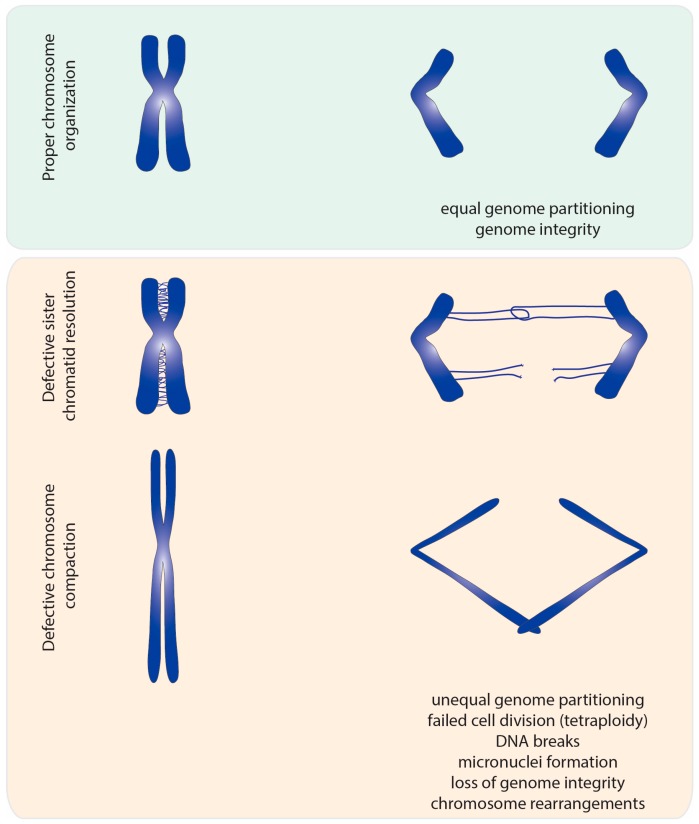
Segregation errors observed upon topoisomerase II malfunction. Impairment of topoisomerase II function results in lack of sister chromatid resolution and insufficiently compacted chromatin. In normal mitosis, sister chromatid intertwines are almost fully resolved at metaphase to ensure faithful genome segregation. Lack of topoisomerase II results in extensive entanglements between sister chromatids that persist during anaphase. Such entangled DNA threads not only hinder equal chromosome segregation, but may also lead to generation of breaks in DNA. In addition, chromosomes deprived of topoisomerase II display lowered levels of compaction. Under-compacted chromosomes are also prone to segregation errors during anaphase and cytokinesis, as the cleavage furrow may trap long and missegregated chromatids.

This catenation/decatenation activity is crucial to maintain the topological state of both interphase and mitotic chromosomes. Although the role of topoisomerase II is not limited to mitosis [4], inhibition of topoisomerase II has the most dramatic consequence during nuclear division. Failures in resolving sister chromatid intertwines and/or compacting chromosomes lead to extensive DNA bridges when chromosomes attempt to segregate to opposite poles of the cell (Figure 2). These errors can result in asymmetric chromosome segregation, followed by loss/gain of genome content (e.g., segmental aneuploidy). Additionally, chromatin bridges may be trapped by the cleavage furrow at the end of cell division. This can cause extensive DNA damage, and thereby potentiates the accumulation of mutations and chromosome rearrangements. Thus, efficient chromosome assembly requires major changes in chromatin organization: sister chromatid resolution, chromosome individualization and chromosome compaction. Such changes induce concomitant modulation of chromosomal mechanical properties, required to withstand the dynamic processes of nuclear division. As described below, topoisomerase II is a critical enzyme for all these processes.

## 2. Topoisomerase II and Sister Chromatid Resolution

Two replicated DNA molecules are extensively topologically entangled with each other, mainly as a consequence of the replication process. Copying the DNA molecule requires unwinding of its double helix, leading to the accumulation of helical tension ahead (positive supercoils) and behind (negative supercoils) the replication fork. Resolution of these topological constrains can be achieved either by direct removal of supercoils (mediated both by topoisomerase I and II) or by rotation of the replication fork. The latter results in intertwines between the two newly replicated strands. In eukaryotes, topoisomerase II is the only enzyme capable of resolving these catenations between DNA molecules. Timely resolution of sister chromatid intertwines is especially important during mitosis to ensure physical individualization of sister DNA molecules (and also neighboring chromosomes), that need to be distributed between the two daughter cells. Cells lacking topoisomerase II undergo a faulty anaphase with extensive chromatin bridges due to unresolved topological links [5,6,7]. Additionally, loss of resolution, particularly at the centromeric region, impairs efficient bipolar attachment to the mitotic spindle, thus increasing the extent of mitotic defects upon topoisomerase II depletion [8].

Most catenations linking DNA molecules are resolved during replication or before mitotic entry, as revealed by measurements of the frequency of catenated circular mini-chromosomes throughout the cell cycle [9]. Moreover, several lines of evidence support that the G2/M checkpoint is somehow sensitive to the levels of DNA catenation, and is able to prevent mitotic entry in cells with compromised topoisomerase II activity [10,11,12,13,14]. What is sensed by this putative “topology checkpoint” is not clear. This checkpoint is unlikely to directly measure the presence of catenation, but possibly indirect effects that an excessive topological stress may induce in the DNA molecules. Consequently, cells enter mitosis with a considerable amount of unresolved catenations that need to be resolved during the process of nuclear division [9,15,16,17]. 

Prophase is a crucial time for sister chromatid resolution. Analysis of the kinetics of sister chromatid resolution has been recently studied in great detail, either by using live cell imaging approaches, or methods that specifically label individual sister chromatids [15,16]. These studies revealed that the vast majority of mitotic entanglements between sister chromatids are resolved by the end of prophase, allowing clear individualization of two separate chromatid axes, a process dependent on topoisomerase II activity [15,16]. Interestingly, this topoisomerase II-dependent individualization of sister chromatids starts already in early prophase, and coincides in time with chromosome condensation [15].

These, and other studies, led to the idea that sister chromatid resolution would be mostly completed at early mitotic stages through a linear and gradual process. In contrast to this notion, recent findings provide a critical change in our understanding of chromosome resolution during mitosis by highlighting the reversibility of this process [18,19,20] (discussed in detail in Section 5.4 and Section 5.5). These results highlight that previously separated DNA molecules are able to re-intertwine as a consequence of topoisomerase II action. This implies that during metaphase, catenations are not only resolved, but they can arise de novo. Therefore, the number of catenations during metaphase results from a net effect of this bidirectional process. Tight regulation of topoisomerase II activity is thus required to ensure that chromosomes display enough entanglements to ensure the right compaction and mechanical stiffness (discussed below), which is still compatible with their efficient resolution in late anaphase.

Anaphase segregation offers the “last chance” for the resolution of sister chromatid intertwines. Several lines of evidence support that anaphase chromatids can still be connected with each other by residual catenation, also known as ultra-fine bridges (UFBs). Major problems in sister chromatid resolution can lead to large DNA bridges, easily visualized by common DNA dyes. In contrast, ultra-fine DNA bridges, prevalent in unperturbed mitosis, cannot be visualized by chromatin dyes [21]. These UFBs are coated by specific helicases, namely Bloom syndrome protein (BLM) or Plk1-interacting checkpoint helicase (PICH) [22,23]. Some of these bridges arise due to under-replication of the DNA, particularly at sites whose replication itself is problematic, due to repetitive sequences or secondary structures (e.g., centromeres, telomeres, fragile sites) [24,25,26,27,28]. However, the most prevalent bridges detected in unperturbed cells actually arise from incomplete decatenation, and are most prominent at the centromeric regions [22,23,24,29]. Topoisomerase II is found preferentially at centromeres already during metaphase chromosomes [30,31], probably reflecting a high level of entanglements in this region. The frequency of ultrafine bridges decreases as anaphase progresses, implying these are actively resolved during these late mitotic stages [22,23,29]. In accordance, PICH was recently shown to recruit and stimulate topoisomerase II activity to UFBs [32].

## 3. Topoisomerase II and Chromosome Compaction

Compaction of interphase chromosomes into rod-shaped structures is a crucial step in ensuring faithful DNA division [33,34]. By condensing DNA, cells achieve spatial compaction necessary in a limited cytoplasmic space, and favor biorientation of sister chromatids for even genetic material division.

The idea that topoisomerase II could be involved in chromosome compaction stems from classical studies that revealed that this enzyme is one of the most abundant non-histone proteins found on mitotic chromosomes. Early research on chromosome structure showed that after histone extraction, chromosomes on electron microscopy images take shape of loops of DNA attached to a dense scaffold [35,36,37]. Composition analysis of the observed scaffold revealed that the major components were topoisomerase II and condensin complexes [38,39]. This discovery led to the proposal that these proteins would form a scaffold within the chromosome axis to which radial chromatin loops would be anchored, thereby compacting mitotic chromatin. Since those observations, the presence of a rigid protein scaffold at the axis of chromosomes has been highly debated (some of the discussion reviewed in [34,40]). Of particular relevance against the idea of a highly stable stiff scaffold for DNA loops, was the finding that both condensin I and topoisomerase II display a highly dynamic association with chromatin [41,42,43,44]. Such dynamic behavior would, instead, be more compatible with an enzymatic action of topoisomerase II underlying mitotic chromosome assembly (discussed below).

The extent to which topoisomerase II contributes to chromosome compaction has been difficult to establish, as various research done in different model systems present conflicting results. Studies using topoisomerase II inhibitors invariably report that, in addition to severe chromosome segregation defects, chromosome compaction is also impaired [6,45,46,47,48,49]. However, it has been largely argued that these results could stem from unspecific effects of the inhibitors, or to the fact that several of these inhibitors trap topoisomerase II onto the DNA and/or induce DNA damage. For example, a widely used topoisomerase II inhibitor, ICRF-193, locks the enzyme in a DNA-bound “closed-clamp” conformation [48,50]. Upon treatment with another inhibitor, teniposide (VM-26), the enzyme forms a covalent intermediate with a cleaved DNA double strand [51]. However, long chromosomes were also observed upon treatment with a wide range of topoisomerase II inhibitors, including catalytic inhibitors that do not stabilize enzyme–DNA complexes [49,52]. Thus, defects in chromosomal longitudinal compaction seem to be a consistent trait upon topoisomerase II inhibition, regardless of the mechanism of action of the inhibitor.

Direct removal of the protein using genetic approaches or RNA interference should circumvent potential caveats arising from the use of inhibitors. Indeed, in several model systems, inactivation of topoisomerase II often leads to defects in chromosome compaction, although the extent of these defects varies significantly among different studies. 

Genetic studies in *Saccharomyces cerevisiae* failed to detect significant changes in chromosome compaction in mutants for topoisomerase II [53]. These studies were based on FISH measurements of the rDNA locus, and thus, may reflect a particular organization of these chromosomal regions. By contrast, direct measurements of the distance between two distal chromosomal sites support that topoisomerase II is required for linear condensation in budding yeast [54]. Similar studies in *Schizosaccharomyces pombe* further support the role of topoisomerase II in chromosome compaction [5,55].

In metazoans, cells lacking topoisomerase II display abnormal chromosome morphology, particularly along their longitudinal axis. However, the extent of these defects is highly variable across various studies, ranging from very mild defects or delayed compaction kinetics, to severe morphological alterations. These include studies in plants [48], *Caenorhabditis elegans* [56], *Drosophila melanogaster* [57,58,59], chicken cells [60,61], and human cells [62,63,64].

In contrast to yeast and invertebrates, vertebrate cells have two topoisomerase II isoforms: alpha (α) and beta (β). Both isoforms are required for sister chromatid resolution, and depletion of a single isoform gives rise to segregation defects [62]. Although some reports suggest that topoisomerase II α and β are partially redundant for mitotic chromosome condensation [62], depletion of topoisomerase II α alone was shown to compromise shortening of the longitudinal axis [61,65]. Moreover, hypercompaction of chromosomes, induced by prolonged mitosis, is abolished in the absence of topoisomerase II α [66].

Studies in vitro, where sperm chromatin is incubated with *Xenopus laevis* mitotic extracts, have also provided a valuable tool to dissect the mechanisms of mitotic chromosome assembly. Topoisomerase II was shown to be absolutely required for the condensation of interphase nuclei into discrete chromosomes in these in vitro systems [67,68]. More recently, a minimalistic approach aimed to identify the components present in a mitotic extract that are sufficient to reconstitute phenotypically normal mitotic chromosomes from interphase *Xenopus* sperm chromatin, in vitro. This approach has also highlighted topoisomerase II as one of the six factors required for chromosome assembly in this assay [69]. It nevertheless remains to be addressed if topoisomerase requirement relies exclusively on disentangling DNA, or also an active role in chromatin compaction. Indeed, blocking topoisomerase II activity in these extracts, once chromosomes had already formed, does not lead to chromosome disassembly, arguing that this enzyme is not required to maintain the compacted state of chromosomes [68].

Despite the evidence supporting topoisomerase II role in chromosome compaction, several discrepancies still make this a highly controversial issue. These discrepancies may be due to specific assays, particularly the extent of topoisomerase II inhibition or the experimental layout. As mentioned above, topoisomerase II inhibition is known to block mitotic entry [10,11,12,14]. Thus, severe reduction of topoisomerase II levels or activity leads to a drastic decline in the mitotic index [11,14,57,70]. This precludes the analysis of complete absence of topoisomerase II during mitosis, unless cells are artificially forced to bypass the G2/M checkpoint (with the confounding effects this override may impose). Inhibition or removal of topoisomerase II prior to mitotic entry has an additional caveat: even if cells are able to bypass cell cycle checkpoints and enter mitosis, it is conceivable that the excess of DNA catenation present in topoisomerase II-depleted chromosomes may alone mask, or modify, any compaction defect existing on those chromosomes.

Acute inhibition of topoisomerase II thus offers a powerful approach by which one can follow the immediate changes in chromosome condensation levels, as metaphase-timed inhibition can be triggered experimentally. Indeed, treatment with topoisomerase II inhibitors in metaphase cells, with pre-assembled chromosomes, leads to rapid chromosome decompaction, particularly elongation of the longitudinal axis [7,20,49,71]. To date, such metaphase-specific perturbations have only been performed using small molecule inhibitors. Recent developments on experimental tools for acute protein inactivation/degradation should soon clarify this controversial issue.

How topoisomerase II could mediate shortening of chromosomal axis remains unknown. It is not clear whether or not the effect on chromosome compaction results from topoisomerase catalytic activity or, alternatively, a non-enzymatic role of this protein. Classical studies highlight the abundance of topoisomerase II on metaphase chromosomes, which argued for a more structural role [38]. Additionally, differential inhibitor response further reasoned that catalytic activity of topoisomerase II is not required for metaphase chromosome assembly [72].

Non-enzymatic roles for topoisomerase II have been described to contribute to various aspects of mitotic fidelity, including the recruitment of mitotic regulators to centromeres and checkpoint signaling, and occur through the C-terminal domain of this enzyme [73]. However, evidence supports that the role of topoisomerase II in chromosome compaction involves its enzymatic catenation activity. As mentioned above, topoisomerase II binds dynamically with mitotic chromatin [44,74]. Importantly, this dynamic behavior is abolished if cells are treated with VM-26 [74]. This drug stabilizes covalent catalytic DNA intermediates of topoisomerase II, after double-stranded DNA cleavage [75]. Thus, these findings imply that the dynamic behavior displayed on mitotic chromosomes by the entire chromosomal-bound pool arises from engaging into cleavage reactions. More direct proof arises from the findings that in human cells, topoisomerase II α displays chromosome compaction defects that are rescued by a wild-type version of topoisomerase II, but not by a catalytic mutant version (K662R) [66]. If so, how can catenation/decatenation reactions dictate the state of chromosome compaction, particularly along the longitudinal axis? A potential explanation is that the presence of extensive catenations linking sister DNA molecules could alone impede the assembly and compaction of mitotic chromosomes. Alternatively, maintenance of chromosome morphology may require a more active role of topoisomerase II throughout mitosis. A possible model is that topoisomerase II is introducing self-entanglements in the DNA molecules, and thereby promote shortening of axial length [3,76].

Chromosome shortening mediated by intramolecular entanglements would predict the formation of knots within the same DNA molecule. Although these products are readily observed in circular naked DNA [77,78,79], their possible formation and presence in the context of chromatin has been largely questioned. Recent findings now provide evidence for the existence of intramolecular DNA knots in a variety of yeast circular minichromosomes [80]. Other evidence for chromosome self-entanglement has mostly arisen from biophysical studies, and will be discussed below.

## 4. Topoisomerase II and Biophysical Properties of Chromosomes

Another important aspect of creating mitotic chromosomes is to ensure the right mechanical properties of chromatin, to sustain DNA integrity when chromosomes are subjected to the pulling and pushing forces imposed by the mitotic spindle, cytoplasmic drag, and other factors. The regulation of topological entanglements within a chromatin network provides a means for changing physical properties of chromosomes, such as stiffness, elasticity, bending rigidity, and physical dimensions, among others. Thus, topoisomerase II has been proposed to also contribute to mitotic chromosome structure by modulating the biophysical properties of chromosomes. This idea was first raised after observations that topoisomerase II is able to decrease elastic stiffness of isolated newt mitotic chromosomes [76]. Mitotic chromosomes become more elastic after treatment with topoisomerase II α and ATP, which is interpreted as relaxing DNA entanglement within a chromosome due to topoisomerase activity. These experiments led to the proposal that the number of self-entanglements within the chromosomes, imposed by topoisomerase II, would influence the biophysical properties of mitotic chromosomes. As topoisomerase II is able to both entangle and disentangle DNA, it would provide a way to modulate stiffness and elasticity of chromosomes throughout mitosis. This idea is further supported by lab-on-a-chip microfluidics approaches, in which manipulation of topoisomerase II activity lead to drastic changes in the shape of protease-treated mammalian chromosomes [3]. In these studies, addition of topoisomerase II to isolated chromosomes leads to a significant decrease in chromosome length and an increase in roundness, compatible with the excessive formation of catenations in the DNA network, within individual chromatids.

The physical properties of chromosomes are of utmost importance at the centromeric region. Many regulatory processes that ensure mitotic fidelity (e.g., spindle assembly checkpoint and error-correction) depend directly or indirectly on the formation of tension (reviewed in [81,82]). Thus, if topoisomerase II controls chromosomal stiffness, inhibition of this enzyme would be expected to affect mitotic progression. In accordance, several studies report that topoisomerase II removal triggers a metaphase arrest that delays anaphase onset [83,84,85]. It has been argued that such delay reflects the presence of a “topology checkpoint” [73,86]. It is nevertheless conceivable that a compromised structure on the pericentromeric chromatin may alone perturb microtubule–kinetochore attachments, and thereby trigger the spindle assembly checkpoint by conventional means. In agreement, yeast mutants for topoisomerase II display extensive chromatin stretching at the centromeric region and an altered sensitivity to tension-dependent chromosome detachments, suggesting that topoisomerase II determines the tensile properties of centromeric chromatin [87].

## 5. Regulation of Topoisomerase II Activity: Guidance by the Structural Maintenance of Chromosomes (SMC) Complexes

### 5.1. The Directionality Problem

As outlined above, topoisomerase II is actively engaged into shaping mitotic chromosomes throughout the process of nuclear division. It promotes the disentanglement of sister DNA molecules, required for efficient chromosome resolution. In parallel, this enzyme contributes to the compaction of individual chromatids, possibly by introducing self-entanglements. This dual function raises a strong directionality problem. How can topoisomerase efficiently remove catenations in trans and thereby resolve sister DNA intertwines, concomitantly with introducing entanglements in cis to compact/confer rigidity to mitotic chromatin? In other words, how does topoisomerase II distinguish between strands from the same DNA molecule from the sister strand?

The activity of topoisomerase II throughout mitosis is regulated by post-translational modifications, such as sumoylation and phosphorylation. Mitosis-specific phosphorylation of topoisomerase II was shown to increase the catalytic activity of this enzyme [88,89]. Conversely, sumoylation of topoisomerase II is necessary for its localization to centromeres in metaphase, leading to decreased decatenation activity of topoisomerase II [31,90,91,92]. This modification was shown to be crucial for correct chromosome segregation [31,93,94]. However, to date, none of the modifications that regulate of topoisomerase II enzymatic activity have provided mechanisms to solve the directionally issue. 

One possible mechanism to ensure efficient decatenation could rely on an innate bias of topoisomerase II, which could recognize DNA topology to find an appropriate substrate. It was proposed that topoisomerase II could somehow recognize juxtapositions of two DNA helices, which are common in knots and catenanes. Such substrate selection could drive the preferential removal of topological links, thereby simplifying the topology (as reviewed in [95]). This intrinsic ability of type 2 topoisomerases fits with the observations that these enzymes are able to remove topological links (knots, catenanes) from DNA, in vitro, even if the initial DNA substrates already contain a very small proportion of linked DNAs [96]. The capacity of resolving topological links below equilibrium was most prominent for topoisomerase IV from *Escherichia coli*, but weaker for human or *Drosophila* topoisomerase II [96], suggesting that intrinsic topology simplification may not be sufficient to ensure decatenation. Other mechanisms must then guarantee efficient bias towards decatenation of DNA molecules.

Structural maintenance of chromosomes (SMC) complexes are emerging as critical players in regulating the extent and/or the direction of topoisomerase II reactions. SMC complexes are major organizers of mitotic chromosomes [33,97,98]. Most notably, SMC complexes in mitosis assist in obtaining a condensed state of chromatin, mechanical properties necessary for segregation, and sister chromatid cohesion. The exact mechanisms by which topoisomerase II cooperates with SMC complexes in shaping mitotic chromosomes are yet to be understood. Below we discuss speculative models for how SMC complexes could influence the outcome of topoisomerase II-mediated decatenation/re-catenation reactions.

### 5.2. Bacterial SMCs

The most direct evidence for functional interactions between topoisomerases and SMC proteins was observed in prokaryotes. MukBEF complex, the SMC complex found for example in *E. coli*, plays an essential function in chromosome compaction and segregation [99]. Its MukB subunit was shown to physically interact with ParC subunit of topoisomerase IV (a type 2 topoisomerase) and this interaction increases the knotting and supercoiling relaxation activities of topoisomerase IV [100,101,102]. Additionally, MukBEF is responsible for localizing topoisomerase IV to *ori* regions [103]. ParC and MukB interaction was shown to be necessary to promote decatenation of freshly replicated *ori*s, further supporting that MukBEF promotes decatenation activity of topoisomerase IV in vivo [104]. Besides its role in chromosome segregation, topoisomerase IV was also proposed to aid MukBEF complex in chromosome condensation. Recent data suggest that topoisomerase IV can stabilize the MukB homodimer on the DNA, and thus enhance compaction of DNA [105]. Interestingly, the same study shows that even catalytically inactive ParC subunit of topoisomerase IV is able to promote MukB driven compaction. Thus, the interaction between topoisomerase IV and MukBEF complexes may serve as a scaffold for efficient DNA compaction and organization.

In contrast to MukBEF, another member of prokaryotic SMC complexes, called SMC-ScpAB, present in *Bacillus subtilis*, displays a different behavior in regards to topoisomerase IV. To date, SMC-ScpAB was not reported to physically interact with topoisomerase IV subunits. Moreover, in *B. subtilis,* the functional cooperation between topoisomerase IV and the SMC complex is not necessary to resolve newly replicated *oris* and the SMC complex alone is sufficient to perform efficient *ori* segregation [106]. However, overexpression of topoisomerase IV in *B. subtilis* is able to partially rescue the segregation and compaction defects present in SMC mutants [107], suggesting a partial overlap in functions of the two proteins. 

### 5.3. SMC5/6

The SMC5/6 complex is known mostly for its DNA repair activity and in aiding replication [108]. SMC5/6-topoisomerase II interplay seems to be important for error-free replication. Yeast SMC5/6 was observed to accumulate at sister chromatid intertwine sites, and topoisomerase II was needed for the resolution of those links [109,110]. Similarly, human topoisomerase II was shown to physically interact with SMC5/6 subunits, in order to resolve topological entanglements during replication [111]. Although the function of this complex is mostly outside of mitosis, its impact on chromosome organization also influences mitotic chromosome morphology. Direct evidence for the importance of SMC5/6 to mitotic chromosome assembly is based on observations that human cells lacking SMC6 or SMC5 display defective chromosome morphology and faulty mitotic localization of topoisomerase II or condensin [112,113]. Whether or not the SMC5/6 complex is present on mitotic chromatin has been controversial. Whereas some studies reveal it is virtually absent from mitotic chromosomes [112], others report an enrichment at pericentromeric regions, at the chromosomal axis, and at DNA bridges caused by topoisomerase II inhibition [113,114]. It thus remains unclear if this complex is actively shaping chromatin during mitosis.

### 5.4. Cohesin—The Resolution Blocker

Cohesin is loaded onto chromosomes in the G1 phase of the cell cycle to establish sister chromatids cohesion during replication [98,115,116]. In prophase, cohesin is removed from chromosome arms, but it is retained at the centromeric region until the anaphase onset, giving rise to the classical, X-shaped mitotic chromosomes morphology [115,117]. As described above, centromeres of early anaphase are often connected by ultra-fine bridges, which are later resolved with help from topoisomerase II and specialized helicases (i.e., BLM and PICH). It is hypothesized that centromeric regions are prone to such bridges due to presence of cohesin, that keeps sister chromatids close together until anaphase, preventing earlier removal of catenations [29,118,119]. Studies in yeast and HeLa cells implied that cohesin release is absolutely necessary to enable the removal of persistent catenations by topoisomerase II, and the presence of cohesin locally prevents topoisomerase II decatenation activity [29,118]. If so, resolution of cohesin-rich regions can only fully resolve after anaphase onset, once cohesin is completely removed, leading to temporary chromatin threads. In agreement, chromosomes with increased levels of cohesin at chromosomal arms are still able to segregate, but display significant chromatin bridges or stretching during late mitosis [120,121,122]. Although cohesin does hamper sister chromatid decatenation, this unlikely presents a full block. Measurements of sister chromatid resolution based on differential labelling of individual sister chromatids reveal that cells that retain cohesin along chromosome arms in prophase are still able to resolve sister chromatids to a significant extent, enabling their visualization, despite the high levels of cohesin [15].

**Figure 3:**
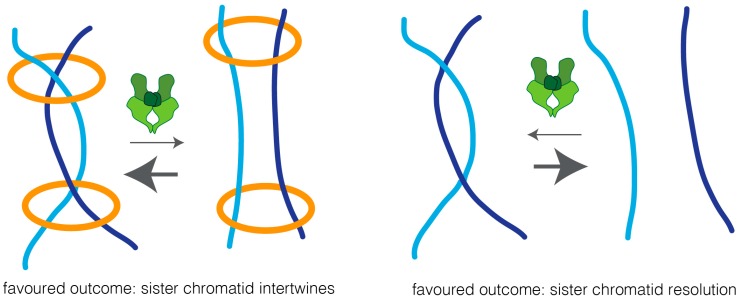
A proximity model for the regulation of topoisomerase II reactions by cohesin. Topoisomerase II undergoes bidirectional reactions, promoting both the resolution and re-catenation of DNA molecules. The cohesin complex (in orange) binds together two DNA molecules (in light blue and dark blue respectively), bringing them in close proximity. Such closeness can increase the chances of topoisomerase II-driven catenation of those strands, and thereby shifts the equilibrium towards the catenated state. Removal of cohesin results in physical separation of DNA molecules, rendering them unlikely to re-entangle once topoisomerase II separates them. Thus, upon cohesin removal, the equilibrium shifts, favoring sister chromatid resolution.

Two possible models could explain how cohesin impairs sister chromatid resolution: cohesin may work as a physical barrier, and thus prevent the accessibility of topoisomerase II to the intertwined chromatin region, thereby preventing efficient decatenation. Alternatively, cohesin may keep sister chromatids in such close proximity that biases topoisomerase reactions towards the intertwined state (Figure 3). In support of the latter is the finding that overexpression of topoisomerase II leads to sister chromatid re-entanglement, in a manner that depends on cohesin [19].

It has been extensively argued whether the presence of residual catenation in mitotic chromosomes is simply a by-product of DNA decatenation enzymology or, alternatively, this residual catenation may play a direct role in chromosome segregation. Before the identification of the cohesin complex, persistent DNA catenations were proposed as the mechanisms that keeps sister chromatids together [123]. Removal of cohesin has since been demonstrated to be sufficient to fully abolish sister chromatid cohesion [7,124], and cohesin is thus the principal cohesive factor. This, however, does not exclude that physical entanglements between sister chromatids (protected by cohesin complexes) could also serve as an additional cohesion mechanism aiding to keep sister chromatids together, and favoring biorientation [118,125,126,127,128]. Clarifying the exact contribution of these DNA links for sister chromatid cohesion is, nevertheless, a virtually impossible task, as their presence and resolution depends on cohesin dynamics [19,29,118].

### 5.5. Condensin—The Guiding Complex

Condesins are key complexes required to establish and maintain mitotic chromosome organization (for a recent review, see [33,129]). Condensin has been proposed to mediate chromosome condensation [69,130,131] concomitantly with providing mitotic chromatin the right physical properties [20,42,132,133,134].

In addition to the proposed structural role, condensin was shown to facilitate sister chromatid separation, as lack of condensin in multiple organisms led to impaired segregation in anaphase [9,20,132,135,136,137].

The exact mechanism of how condensin is contributing to DNA resolution is not fully understood. Unlike topoisomerase II, condensin complexes cannot (de) catenate DNA molecules. Thus, it is highly probable that condensin is cooperating with topoisomerase II (directly or indirectly) to achieve this goal. Various mechanisms have been hypothesized to establish how condensin could aid in sister chromatid resolution. Initial studies propose that condensin directly enhances topoisomerase II enzymatic activity [135,138]. However, other studies failed to detect similar topoisomerase activation, suggesting that condensin promotes sister chromatid resolution by other means [9,139]. It has long been speculated that condensin could somehow provide directionality for topoisomerase II activity. Although very attractive, this model lacked experimental validation of its two major premises: (1) topoisomerase II is indeed engaged into bidirectional reactions in metaphase chromosomes; (2) condensin complexes direct this reaction in favor of decatenation.

Recent studies provide evidence in support of a directionality model in which condensin emerges as a critical complex to favor sister chromatid resolution, rather their re-intertwine [18,19,20]. The first evidence that topoisomerase II is indeed able to catalyze the catenation/decatenation reactions in a bidirectional manner, in vivo, arises from studies in budding yeast, using circular minichromosomes. Metaphase-specific de novo re-catenation, i.e., re-intertwining of previously separated DNA-strands, could be observed either by the disruption of spindle forces [18] or overexpression of topoisomerase II [19]. Minichromosomes are, nevertheless, under different topological constrains, due to their circular nature. Moreover, they are much smaller when compared to native mitotic chromosomes. Whether or not these finding would translate to large, linear chromosomes was unknown.

Our recent studies in *Drosophila* embryos demonstrate that acute removal of condensin I from pre-assembled chromosomes leads to extensive re-intertwining of previously separated sisters, mediated by topoisomerase II [20]. In contrast, metaphase-specific inactivation of topoisomerase II leads to minor segregation errors. These findings indicate that in metaphase chromosomes, the majority of catenations are efficiently resolved. However, upon inactivation of condensin I complex, specifically during metaphase, previously resolved sisters are able to re-intertwine, in a topoisomerase II-dependent manner. Such de novo intertwines that arise during metaphase lead to drastic defects in chromosome segregation, marked by massive anaphase bridges. Thus, topoisomerase II is engaged in both catenation and decatenation reactions in large linear chromosomes. Importantly, these results highlight the need for condensin I complex in continuously providing a bias in the direction of these reactions. All together, these results imply that condensin modulation of topoisomerase II activity is not catalytic, but rather imposes strong bias towards decatenation, which is absolutely necessary to prevent topoisomerase II from introducing excessive de novo entanglements. Constant guiding activity of condensin I is therefore required throughout metaphase. Such a dynamic view for the sister chromatid resolution process may thus explain the rapid turnover behavior displayed by both condensin I and topoisomerase II in vivo [41,42,43,44].

The exact mechanism by which condensins direct topoisomerase II reactions are not fully understood, and several mechanisms may be envisioned. Initial studies in the Aragon lab propose a model where this cooperation relies on condensin I ability to change the topology of the DNA molecule [18,140]. Condensin is able to introduce positive supercoiling in vitro [141,142,143], and positively supercoiled DNA was shown to be a preferred substrate for the decatenation reaction of topoisomerase II on yeast circular chromosomes [18]. Thus, by introducing positive supercoiling in the DNA molecule, condensin could bias the direction of topoisomerase II reaction, and thus explain how topoisomerase II and condensin could functionally cooperate to achieve DNA resolution. Surprisingly, recent data suggest that condensin compacts DNA at the same rate, independently of the topological state of this DNA (uncut, nicked, positively, or negatively supercoiled) [144]. Thus, unless condensin displays a different mechanism for sister chromatid resolution, condensin action, in vivo, may not rely on the introduction of supercoils. Moreover, given that de novo intertwines are able to arise from previously decatenated DNA strands in a topoisomerase II-dependent manner, modulation of DNA topology to alter substrate preference is clearly insufficient to explain the observed reversibility of the decatenation reactions.

**Figure 4:**
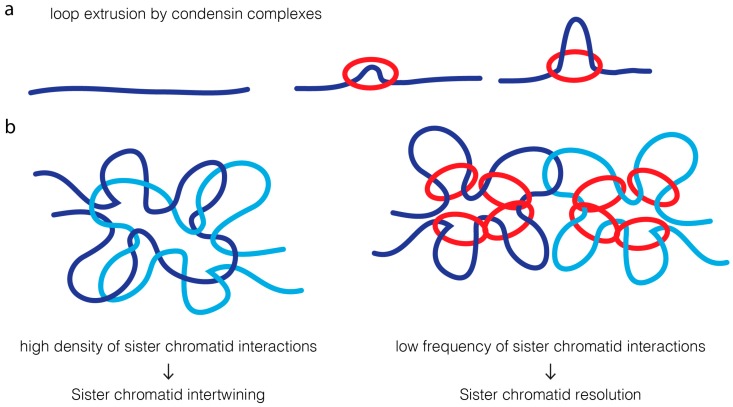
A contact probability model for condensin-directed topoisomerase II reactions. (**a**) Condensin complexes are proposed to create transient loops in the DNA molecule through an extrusion mechanism: binding to a single DNA locus is followed by progressive pulling of DNA strands (dark blue strand) through the condensin ring (red circle), extending the size of the created chromatin loop; (**b**) Proposed model for condensin role in directing the decatenation activity of topoisomerase II. Strands of replicated sister chromatids are initially heavily entangled. In such state, one copy of DNA molecule (light blue strand) has a very high likelihood of its sister DNA (dark blue strand). Topoisomerase II is therefore likely to catalyze re-intertwining intermolecularly, leading to an extensive degree of re-catenation between two sister chromatids. Condensin activity favors the creation of intramolecular chromatin loops, and thereby decreases the contact probability between different DNAs. The physical separation of the two molecules makes them less likely to be re-intertwined together, creating a bias towards intermolecular decatenation.

A much simpler model is based on the modulation of contact probability [19,20] (Figure 4). Condensins have been proposed to extrude chromatin loops [145,146,147,148,149,150]. An attractive feature of the loop-extrusion model is that it would ensure that condensin promotes long-distance interactions exclusively within the same DNA molecule [145,147]. High density of intrachromatid contacts, in cis, reduces the probability of interaction of two sister DNA molecules. This decrease in probability of contacts between sister DNA molecules could alone reduce the probability of re-catenation by topoisomerase II, favoring their efficient resolution.

**Figure 5:**
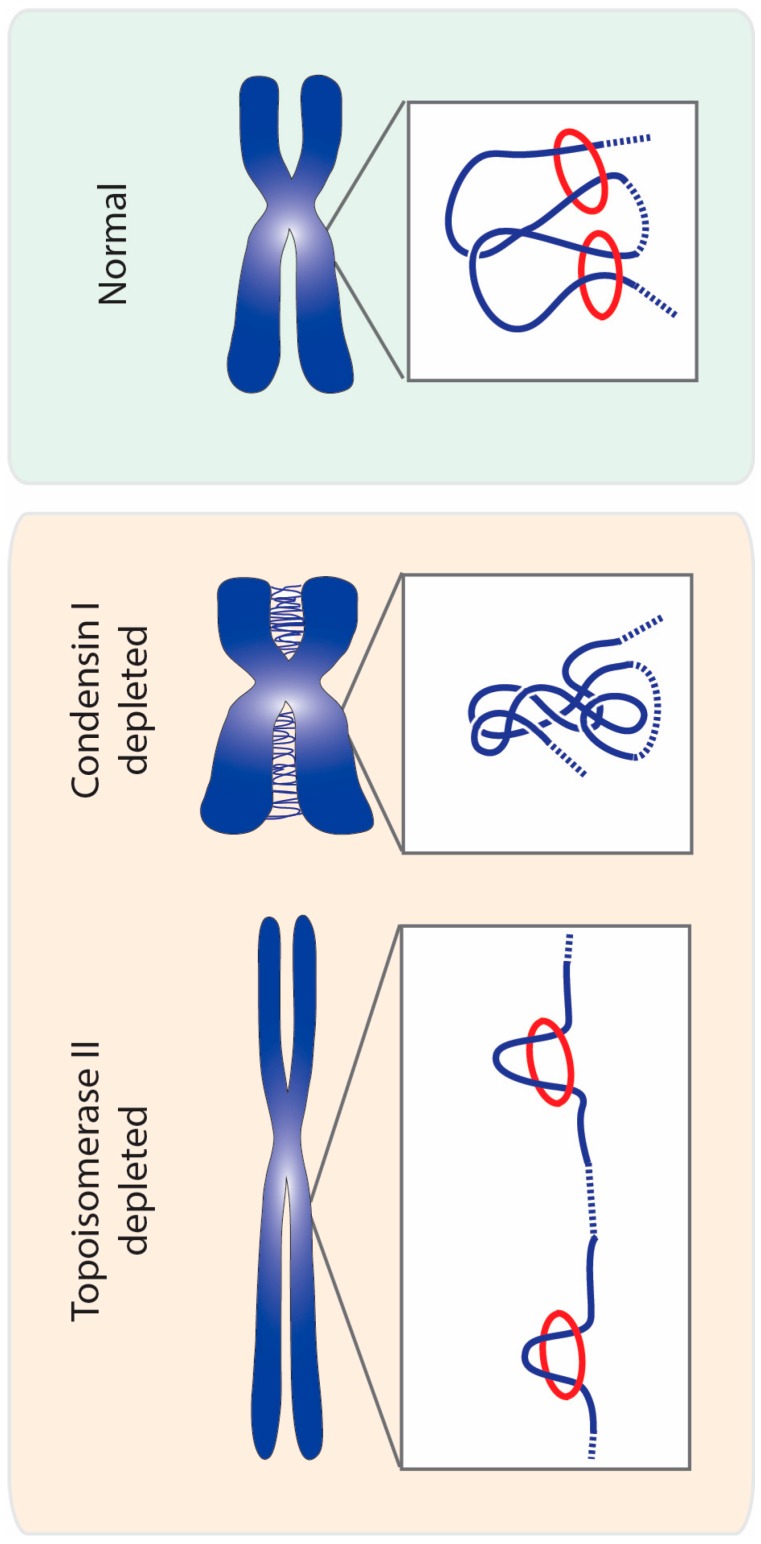
A topology model for chromosome compaction mediated by topoisomerase II and condensin. Proper chromosome compaction may result from a balance of self-catenation, imposed by topoisomerase II, within condensin-mediated DNA loops (condensin depicted as red circles and DNA strands in blue). Self-catenation may work as a loop-stabilizer between adjacent loops, thereby ensuring chromosome compaction. Removal of condensin I from mitotic chromosomes prevents the formation of loops, and consequently allows the formation of excessive chromosome entanglements by topoisomerase II (both intra and intermolecularly). Such massive amounts of chromosomal entanglements may lead to an overall increase in chromosome compaction. In turn, impairing topoisomerase II causes loss of chromosome compaction, especially along the longitudinal plane. This may be explained by the absence of self-entanglements promoted by topoisomerase II action, which prevents loop-stabilization and/or distal intramolecular connections.

In contrast to the cooperative action for condensin and topoisomerase II in sister chromatid resolution, the interplay of these two proteins in chromatin compaction is, by far, much less understood. Phenotypic analysis suggest they have opposing/distinct roles: condensins were proposed to drive lateral compaction, while topoisomerase II induces axial compaction [60,151]. These apparently antagonistic roles in chromatin organization may also be better explained by the modulation of contacts imposed on mitotic chromosomes, dictating topoisomerase II reactions (Figure 5). In wild type chromosomes, condensin-mediated loop instructs topoisomerase II to avoid re-catenation in trans, which may concomitantly promote and regulate the extent of self-entanglements. Active regulation of entanglements within the same DNA molecules would consequently promote chromatin compaction along its longitudinal axis. In the absence of the guiding role promoted by condensins, sister chromatids will entangle randomly, possibly both in cis and in trans. This leads to an excess of catenation between sister chromatids, and potentially, self-entanglements. Excess of intrachromatid intertwines could then promote the shortening of the longitudinal axis and chromatin hypercompaction, as experimentally observed [20,65]. Short and wide chromosomes may be the default state for mitotic chromosomes if faced with hyperactive topoisomerase II with no guidance for its action. In agreement, isolated chromosomes evaluated on a microfluidic device appear shorter and more round if pre-treated with topoisomerase II [3]. Interestingly, hypercompaction has also been observed if condensin is hyperactivated, which in this case, is likely to be mediated by excessive loop formation [152].

Note that the extent of self-entanglement will also depend on external forces applied on chromosomes. Pulling forces of microtubules were shown to favor chromosome decatenation [18]. Addition of topoisomerase II to in vitro purified chromosomes causes relaxation of chromatin if chromosomes are subjected to pulling forces [76], and chromosome shortening in a low force environment [3]. This may thus explain why meiotic chromosomes, which are pulled along their longitudinal axis, disassemble upon condensin inactivation [153], in sharp contrast with their mitotic counterparts. Similarly, centromeric regions in mitotic chromosomes, which are also pulled by the mitotic spindle, undergo extensive distortion upon condensin removal [20,42,132,133].

Removal of topoisomerase II, in contrast, would lead to a drastic decrease in ability to form chromatin self-entanglements necessary to ensure longitudinal compaction. The dynamic behavior of topoisomerase II suggests that this activity may be required constantly, to actively promote shortening of the chromosomal axis. Removal of topoisomerase II may thus lead to chromosome elongation, as continuous self-entanglement does not take place. Additionally, self-entanglements could potentially stabilize condensin-mediated loops, or even stimulate their production.

## 6. Concluding Remarks 

Despite extensive studies on metaphase chromosome architecture, the mechanisms that drive chromatin reshaping throughout every cell division are far from being understood. In contrast to a static view of chromosome organization, the maintenance of mitotic chromosome assembly is emerging as a much more dynamic process, where modulation of DNA topology may account for concomitant sister chromatid resolution and chromosome compaction. In a topology-centric view, it is tempting to speculate that mitotic shape of chromosomes results from a balance of self-entanglements (topoisomerase II-mediated) and chromatin loops (condensin) that drive chromosome compaction. This active chromosome shaping, together with cohesin removal, ensures concomitant sister chromatid resolution, a highly dynamic and reversible process. Chromatin itself has been long assumed to play a rather passive role during mitosis, and chromosomes are usually compared to a “corpse at a funeral: they provide the reason for the proceedings but do not take an active part in them” [154]. The emerging view of a more dynamic chromosome assembly process may soon reveal a more active role for chromosomes in the process of their own segregation.

## Abbreviations

SMC	Structural maintenance of chromosomes
UBF	Ultra-fine bridges
BLM	Bloom syndrome protein
PICH	Polo-like kinase 1-interacting checkpoint helicase
